# INTEREST GROUP SESSION—ORAL HEALTH: COGNITIVE FUNCTION, SOCIAL SUPPORT, AND ORAL HEALTH STATUS AMONG OLDER ADULTS IN THE U.S. AND ABROAD

**DOI:** 10.1093/geroni/igz038.1265

**Published:** 2019-11-08

**Authors:** Bei Wu, Stephen K Shuman, Michele Saunders

**Affiliations:** 1 New York University, New York, New York, United States; 2 University of Minnesota School of Dentistry, Minneapolis, Minnesota, United States; 3 Div of Aging Research & Geriatric Psychiatry, UT Health-San Antonio, San Antonio, Texas, United States

## Abstract

There is an increasing awareness of the importance of oral health and its associated risk factors among older adults. This symposium includes four papers that address cognitive function, social support and oral health problems and symptoms among older adults in the U.S. and China. Lu and his colleagues examined the reciprocal relationship between cognitive function and complete tooth loss Chinese adults age 50+ using the China Health and Retirement Longitudinal study. The results show that there is a reciprocal relationship between these two indicators. The second paper used the Population Study of Chinese Elderly in Chicago (PINE) and examined the associations between tooth/gums symptoms and changes in cognitive function in Chinese older immigrants. The results reveal that having teeth symptoms was associated with a decline in cognitive function. Using the same PINE data, the third paper examined the association between different characteristics of social relationships and the number of oral health problems among U.S. older Chinese adults. Wu and her colleagues conducted a partner-assisted pilot intervention to improve oral health for community-dwelling older adults with either mild cognitive impairment or mild dementia. The results of this 6-month intervention show that persons in the treatment group had more improvement in oral hygiene than those in the control group. Findings from these four papers illustrate that cognitive function, social support, and oral health are interrelated. This symposium highlights the importance of improving cognitive health, social support, and oral health for middle-aged and older adults.

